# Optimized protein extraction protocol from human skin samples

**DOI:** 10.1093/biomethods/bpaf035

**Published:** 2025-05-10

**Authors:** Ana Paula Carvalho Reis, Giovanna Azevedo Celestrino, Talita Souza Siqueira, Milena De Melo Scarano Coelho, Juliana Carreiro Avila, Isabela De Oliveira Cavalcante Pimentel, Leo Kei Iwai, Pritesh Jaychand Lalwani, Vitor Manoel Silva Dos Reis, Kaique Arriel, José Ângelo Lindoso, Gil Benard, Maria Gloria Teixeira Sousa

**Affiliations:** Laboratory of Medical Mycology LIM-53, Division of Clinical Dermatology, Instituto de Medicina Tropical, Hospital das Clínicas, Faculty of Medicine, Universidade de São Paulo, Avenida Doutor Enéas de Carvalho Aguiar, 470, São Paulo, SP, 05403-000, Brazil; Laboratory of Medical Mycology LIM-53, Division of Clinical Dermatology, Instituto de Medicina Tropical, Hospital das Clínicas, Faculty of Medicine, Universidade de São Paulo, Avenida Doutor Enéas de Carvalho Aguiar, 470, São Paulo, SP, 05403-000, Brazil; Department of Clinical Medicine, Laboratory of Cellular, Genetic and Molecular Nephrology, University of São Paulo, School of Medicine, Avenida Doutor Arnaldo, 455, São Paulo, SP, 01246-902, Brazil; Laboratory of Medical Mycology LIM-53, Division of Clinical Dermatology, Instituto de Medicina Tropical, Hospital das Clínicas, Faculty of Medicine, Universidade de São Paulo, Avenida Doutor Enéas de Carvalho Aguiar, 470, São Paulo, SP, 05403-000, Brazil; Laboratory of Medical Mycology LIM-53, Division of Clinical Dermatology, Instituto de Medicina Tropical, Hospital das Clínicas, Faculty of Medicine, Universidade de São Paulo, Avenida Doutor Enéas de Carvalho Aguiar, 470, São Paulo, SP, 05403-000, Brazil; Laboratory of Applied Toxinology (LETA) and Center of Toxins, Immune-Response and Cell Signaling (CeTICS), Butantan Institute, Avenida Vital Brasil, 1500, São Paulo, SP, 05503-900, Brazil; Laboratory of Applied Toxinology (LETA) and Center of Toxins, Immune-Response and Cell Signaling (CeTICS), Butantan Institute, Avenida Vital Brasil, 1500, São Paulo, SP, 05503-900, Brazil; Institute Leônidas e Maria Deane (FIOCRUZ-ILMD), Rua Terezina, 476 Manaus, AM, 69057-070, Brazil; Laboratory of Medical Mycology LIM-53, Division of Clinical Dermatology, Instituto de Medicina Tropical, Hospital das Clínicas, Faculty of Medicine, Universidade de São Paulo, Avenida Doutor Enéas de Carvalho Aguiar, 470, São Paulo, SP, 05403-000, Brazil; Institute of Infectious Diseases Emilio Ribas, Avenida Doutor Arnaldo, 165, São Paulo, SP, 01246-900, Brazil; Institute of Infectious Diseases Emilio Ribas, Avenida Doutor Arnaldo, 165, São Paulo, SP, 01246-900, Brazil; Laboratory of Medical Mycology LIM-53, Division of Clinical Dermatology, Instituto de Medicina Tropical, Hospital das Clínicas, Faculty of Medicine, Universidade de São Paulo, Avenida Doutor Enéas de Carvalho Aguiar, 470, São Paulo, SP, 05403-000, Brazil; Laboratory of Medical Mycology LIM-53, Division of Clinical Dermatology, Instituto de Medicina Tropical, Hospital das Clínicas, Faculty of Medicine, Universidade de São Paulo, Avenida Doutor Enéas de Carvalho Aguiar, 470, São Paulo, SP, 05403-000, Brazil

**Keywords:** skin, proteomics, extraction of proteins, protocol, mass spectrometry

## Abstract

The skin is the largest organ in the body and is the site for a diverse set of diseases. Yet, given the complexity of the cutaneous tissue, there is a limited availability of data in the literature on skin proteomics. Here, we proposed an adapted and optimized protocol for the extraction of proteins from human skin, using a combination of chemical and mechanical lysis approaches. For this, we used of a lysis buffer containing 2% SDS, 50 mM TEAB, and a 1% protease and phosphatase inhibitor cocktail, in addition to Matrix A beads and a FastPrep-24 5G homogenizer. For the characterization of the samples, the obtained proteins were purified and digested using the SP3 method (Single-pot, solid phase, sample preparation), and analyzed by nano liquid chromatography coupled with tandem mass spectrometry. In this way, we were able to identify around 6000 proteins in the skin samples from healthy individuals and patients with the fungal infection sporotrichosis. Our improved methodology could significantly enrich our understanding of skin biology and provide new perspectives for the discovery of biomarkers and therapeutic targets for cutaneous diseases.

## Introduction

The skin is the largest organ of the human body, serving as an interface between the organism and the external environment, with essential functions as sensoring, thermal regulation, and protection against invasive microorganisms, chemical agents or any threat to the organism's homeostasis. It is composed of three distinct layers (epidermis, dermis, and hypodermis), harboring diverse cell types, including melanocytes, endothelial cells, keratinocytes, fibroblasts, and skin-associated immune cells. Once this fine balance is disturbed, various skin disorders arise, resulting in physical impairment and compromise of the quality of life [1–3].

Proteomic studies are essential for understanding the wide range of diseases that affect the skin, from infectious ones such as leishmaniasis, tuberculosis, sporotrichosis, dermatophytosis, and viral diseases, to autoimmune diseases such as psoriasis, systemic lupus erythematosus, and scleroderma [1, 4, 5]. For example, in cutaneous leishmaniasis, proteomics can help identifying biomarkers associated with the immune response and the pathogenesis of the infection. In cutaneous tuberculosis, proteomics can reveal changes in markers for differential diagnosis. Similarly, in sporotrichosis, proteomic analysis can elucidate the interactions between the pathogen and the host's immune system, contributing to the development of new therapeutic strategies. In autoimmune diseases, proteomics can identify changes in proteins that reflect immune dysfunction, providing valuable insights for diagnosis and treatment [4, 5].

Nevertheless, there are few studies exploring the molecular composition of healthy human skin at the protein level. One challenge to be overcome for the identification and quantification of specific proteins is the complexity of the skin structure. High-abundance proteins, as keratin and collagen, can mask the detection of proteins of interest, particularly those that play critical roles in pathological processes and may be present in low abundance. Other difficulties involve the technical procedures for sample processing, particularly tissue separation and homogenization [1–3].

Here, we describe an improved method for extracting proteins from human skin samples and detailed the procedures for their characterization by mass spectrometry, which allowed us to identify approximately 6000 proteins.

## Materials and methods

### Ethics committee

This study was approved by the Ethics Committee of the Hospital das Clínicas of the Faculty of Medicine of the University of São Paulo (HCFM/USP) (CAAE: 65476822.6.0000.0068) and by the Ethics Committee of the Emílio Ribas Infectology Institute (IIER) (CAAE: 65476822.6.3001.0061). All donors provided written informed consent prior to sample collection.

### Reagents and equipment

The main reagents used in this study are listed in Table 1 and the main equipment used is detailed in Table 2.

**Table 1.** Main reagents.

Reagent	Supplier/brand	Catalog no.
Sodium dodecyl sulfate (SDS)	Sigma-Aldrich/Merck	L3771
Triethylammonium bicarbonate (TEAB) 1M	Sigma-Aldrich/Merck	T7408-100mL
Ammonium bicarbonate (AMBIC)	Synth	01B1009.01.AG
Halt Protease and Phosphatase Inhibitor Cocktail (100×)	Thermo Scientific	78440
Dithiothreitol (DTT)	Sigma-Aldrich/Merck	D0632-1G
Iodoacetamide (IAA)	Sigma-Aldrich/Merck	I6125-5G
Pierce™ BCA Protein Assay Kit	Thermo Scientific	23227
Acetonitrile (HPLC/MS Grade)	Sigma-Aldrich/Merck	6.18030
Formic acid (MS Grade)	Sigma-Aldrich/Merck	5.33002
Sera-Mag SpeedBeads (Carboxylated, Hydrophilic)	Cytiva	45152105050250
Sera-Mag SpeedBeads (Carboxylated, Hydrophobic)	Cytiva	65152105050250
Absolute ethanol	Sigma-Aldrich/Merck	459828
Sequencing Grade Modified Trypsin	Promega	V511A
Phosphate-Buffered Saline (PBS) [1×]	Sigma-Aldrich/Merck	P2272-500mL
Ultrapure Water (Milli-Q grade or similar)	*Purification system*	–

*Note:* This table lists the main reagents used in this study.

**Table 2.** Main equipment.

Equipment/type	Supplier/brand	Catalog/model no.
Homogenizer (Bead Beater)	MP Biomedicals	FastPrep-24 5G (116005500)
Tubes with Lysis Beads (Matrix A)	MP Biomedicals	Lysing Matrix A, 2 mL (116910050)
Low Retention Microtubes 1.5 ml	KASVI	K6-1515L
Low Retention Microtubes 0.5 ml	KASVI	K6-0505L
Magnetic Rack (for 1.5 ml tubes)	Promega	MagneSphere 12-pos (Z5342)
Refrigerated Benchtop Centrifuge (≥20 000 *g*)	Thermo Fisher	*Specific model* (e.g. 75002431)
Thermomixer (for 1.5 ml tubes)	Eppendorf	*Specific model* (e.g. 5382000023)
Vacuum Concentrator (SpeedVac)	Eppendorf	Vacufuge plus
Mass Spectrometer	Thermo Fisher Scientific	Orbitrap Exploris 480
Liquid Chromatograph (nanoLC)	Thermo Fisher Scientific	Vanquish Neo
Ion Source	Thermo Fisher Scientific	Easy Flex
nanoLC Analytical Column	Thermo Fisher Scientific	Acclaim PepMap NEO (150 mm x 75 μm, 2 μm)
nanoLC Trap Column	Thermo Fisher Scientific	Acclaim PepMap 100 C18 (20 mm x 75 μm, 3 μm)
FAIMS Pro System	Thermo Fisher Scientific	*Integrated with MS*

*Note:* This table lists the main equipment used in this study.

### Procedure

#### Processing of skin samples

1. Skin fragments are collected using a 4 mm punch, transferred to phosphate-buffered saline (PBS) [1×] solution and frozen immediately in liquid nitrogen, being stored at −80°C until protein extraction. Before initiating the extraction, wash the thawed samples twice with sterile PBS 1× to remove excess blood.

#### Proteins extraction

2. Place the samples in pre-chilled tubes on ice containing lysis beads (Lysing Matrix A) and add 2% [w/v] SDS diluted in 50 mM TEAB buffer [lysis buffer] supplemented with 1% protease and phosphatase inhibitor cocktail for a final volume of 600 µl.
3. Process each tube using a FastPrep-24 5G homogenizer (MP Biomedical) applying six cycles of 20 s at maximum speed. • **Note:** Cool samples on ice for 2 min between cycles to prevent thermal degradation of proteins.
4. After the third cycle, leave the sample on ice for 1 h. After this period on ice, perform the remaining three cycles, totaling six cycles.
5. After all cycles, centrifuge the sample for 10 min at 9000 × *g* and collect the tissue lysate.
6. Wash the lysis beads by adding 300 µl of lysis buffer to the tube; vortex for 1 min and centrifuge for 10 min at 9000 × *g*.
7. Combine the wash supernatant (from the beads) with the tissue lysate and centrifuge at 16 000 × *g* for 10 min and transfer the supernatant to a clean tube. Store in the freezer at −80°C until protein quantification.

#### Protein quantification and concentration

8. The protein concentrations of the samples generated after maceration were determined using the bicinchoninic acid (BCA) method [6, 7], using a kit from Sigma-Aldrich (Sigma, Poole, UK).

#### Enzymatic digestion of proteins for MS analysis

This part of the protocol was formulated by referencing the single-pot, solid-phase-enhanced sample preparation (SP3) protocol by Hughes *et al*. developed for protein digestion [8]. In our optimization, protein reduction and alkylation were performed using the reagents DTT and IAA, respectively, with the aim of reducing costs without compromising the yield of this protocol. The full procedure is carried out in 2 days.

**Day 1:**

**Reduction and alkylation**

9. Start with 50 µg of protein from step 7, (adjusting the final volume to 80 µl with lysis buffer) and reduced with 10 mM DTT (45 min, 30°C).
10. Alkylation was performed with 40 mM IAA (30 min, at room temperature, dark).
11. Excess IAA was quenched with 5 mM DTT (15 min, 30°C).

**SP3 cleanup and digestion**

12. **Bead Preparation:** Hydrophilic and hydrophobic Sera-Mag beads (Table 1) were mixed (1:1), washed 3× with ultrapure water, and resuspended in the original volume.
13. **Protein Binding:** 10 µl of the bead suspension (equivalent to 500 µg) were added to the sample (50 µg protein). Incubated (5 min, 1000 rpm, at room temperature). 100% ethanol (equal volume to the sample) was added and incubated again (5 min, 1000 rpm, at room temperature).
14. **Bead Washing:** Beads were immobilized on a magnetic rack (5 min), the supernatant discarded, and washed 3× with 150 µl of 80% ethanol (incubation 5 min, 1000 rpm; magnetic rack 5 min; discard).
15. **Enzymatic Digestion:** 100 µl of 50 mM AMBIC and Trypsin (Table 1) were added at a 1:50 ratio (enzyme: protein). Incubated in the Thermomixer at 1000 rpm at 37°C overnight.

**Day 2:**

**Peptide recovery**

16. Centrifuge the samples (20 000 × *g* for 5 min) and placed on the magnetic rack (10 min).
17. The supernatant containing peptides was transferred to a new low retention microtube.
18. Dry the samples in a SpeedVac (Table 2).

### Acquisition of proteomic data using nano liquid chromatography tandem mass spectrometry

The obtained samples are resuspended in 0.1% formic acid and analyzed using an Orbitrap Exploris 480 mass spectrometer (Thermo Fisher Scientific, Bremen, Germany) coupled with a nanoLC Vanquish Neo liquid chromatograph (Thermo Fisher Scientific) and an Easy Flex source. The system employed a nanoLC Acclaim PepMap NEO analytical column (150 mm × 75 μm, 2 μm) and a nanoLC Acclaim PepMap 100 C18 trap column (20 mm × 75 μm, 3 μm), both from Thermo Fisher Scientific. The peptide extract (0.5 μg) was eluted in a gradient of 5%–40% solvent B (90% acetonitrile, 0.1% formic acid) over 80 min, with a flow rate of 300 nl/min and an electrospray source operated at 2.1 kV.

The spectrometer, equipped with a FAIMS (Field Asymmetric Ion Mobility Spectrometry) system, performed gas-phase ion separation based on their different mobilities under alternating electric fields. Spectra were acquired in full MS mode with a resolution of 60 000 for MS1, within a mass range of 400–900 m/z, using an isolation window of 4 m/z, totaling 125 windows. The AGC (Automatic Gain Control) was set to 300% for Full MS and 1000% for fragment spectra, both with an injection time of 50 ms. The normalized collision energy was adjusted to 28% and 32%. The acquisition of MS/MS spectra was performed using Data Independent Acquisition (DIA) with compensation voltages set in the FAIMS system at −45 and −60 V, a resolution of 30 000, and a maximum injection time of 50 ms.

For data analysis, the .raw files were converted to.mzML using the MSConvert software (SourceForge Inc). Protein identification was performed using the DIANN program (vs 1.8.1) [9] against the *Homo sapiens* database downloaded from Uniprot in December 2024. The search parameters included trypsin as the enzyme, oxidation of methionine as a variable modification, and carbamidomethylation of cysteine as a fixed modification. The search was conducted with a tolerance of 10 ppm for MS and 0.05 Da for MS/MS.

## Results and discussion

To standardize this protocol, skin fragments from healthy individuals were used. Twelve samples of healthy skin were collected, including six women and six men with an average age of 32 years, range: 22–45 years. Additionally, we evaluated its reproducibility by collecting 19 samples of inflamed, lesioned skin obtained from thirteen women and six men with sporotrichosis (average age: 38 years, range: 21–65 years. Sporotrichosis is a subcutaneous mycosis caused by dimorphic fungi of the genus *Sporothrix*. Transmission occurs through traumatic inoculation of *Sporothrix* propagules into the skin. In humans, sporotrichosis typically manifests as inflammatory lesions in the skin and subcutaneous tissues [10–12].

**Figure 1.**
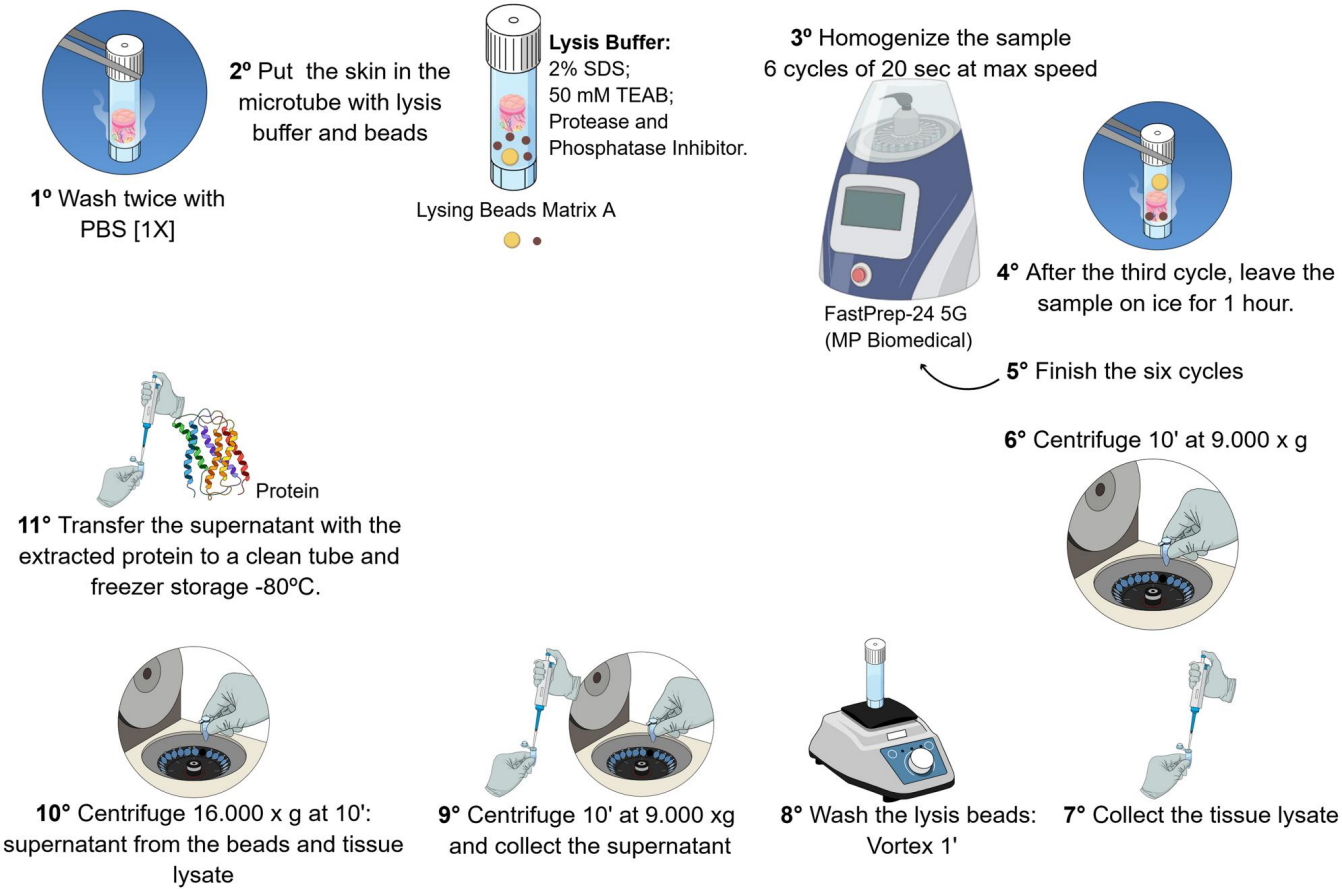
Step involved in optimized protocol for protein extraction of skin sample—workflow sample processing.

In this work, we proposed an optimized protocol for the extraction of proteins from human skin based on existing methods in the literature [13, 14]. Briefly, the modifications that improved the efficiency of protein extraction can be summarized as: (i) increased SDS concentration to 2% and (ii) enhanced mechanical lysis using Matrix A beads, which consist of a garnet matrix and a 1/4 ceramic sphere, presenting varying sizes that facilitate more efficient grinding of the tissue. The cycles of the FastPrep-24 5G homogenizer were also adjusted, reducing the number of cycles and time while increasing the speed to its maximum setting, with the aim of improving tissue breakdown and preventing thermal degradation of proteins. Additionally, the samples were placed on ice between cycles to prevent overheating, which could be detrimental to the proteins. This process is illustrated in Fig. 1.

The total protein concentration in the samples was measured using the Thermo Scientific™ Pierce™ BCA Protein Assay kit, following the manufacturer's instructions. This detergent-compatible kit is based on BCA for the colorimetric detection and quantification of total protein. Following quantification, 50 µg of total proteins from each sample were isolated for subsequent steps, including digestion, purification, and identification by mass spectrometry [6, 7]. As mentioned earlier, we began with a concentration of 50 µg of total proteins and initiated the reduction of these proteins with DTT to break disulfide bonds and alkylation with IAA to covalently modify the SH groups (thiol groups) of cysteine, thereby preventing the formation of new unwanted disulfide bonds [15].

The purification and digestion of proteins were performed using the SP3 method, as described by Hughes *et al*. [8]. This is a more recent approach aimed at minimizing sample loss and streamlining the workflow of proteomic sample preparation. This protocol allows for the simultaneous cleaning of proteins, enzymatic digestion, desalting, and peptide recovery in a single tube [8, 16]. In summary, the extracted proteins were added to the tube with a mass ratio of protein to beads of 1:10 usually 50 µg of protein to 500 µg of beads. We performed a wash with 80% ethanol three times. Following washing, the proteins bound to the beads were digested in a solution containing 50 mM ammonium bicarbonate, along with trypsin in an enzyme-to-protein ratio of 1:50 (1 µg of trypsin to 50 µg of protein) at 37°C, 1000 rpm, overnight. After this incubation period, the peptides were removed from the magnetic beads and dried in a vacuum concentrator before undergoing DIA LC-MS/MS analysis [8]. The median amount of protein extracted from 50 mg of skin tissue for subsequent DIA LC-MS/MS analysis and identification was 2.83 mg (IQR 1.09–3.60). The median yield of protein extraction from the 31 samples was 0.057 mg (IQR 0.02–0.07); individual data are shown in Supplementary Table S1.

Analysis of our sample results revealed that the median number of proteins identified in the control group was 4983 proteins (IQR 4704–5261). In contrast, the patient group with sporotrichosis exhibited higher number of proteins: 5974 (5353–6108, *P *<* *.0006, Mann–Whitney *U* Test). The nonparametric distribution of the dataset was assessed using the Shapiro–Wilk test, and the comparison between the two groups (control vs. patients) was performed using the Mann–Whitney *U* test. Of the proteins identified, 624 were unique to the patient group, while 40 were exclusive to the control group. This information is represented in the Venn diagram (Supplementary Fig. S1).

The increased protein identification observed in the patient group compared to the control may be attributed to the inflammatory processes resulting from the fungal infection, as similarly reported in studies evaluating the proteomic profiles of skin lesions in patients with leishmaniasis and atopic dermatitis [17–19]. In response to tissue injury, the organism initiates a chemical signaling cascade, involving molecular pathways such as NF-κB, which stimulate biological responses aimed at repairing the affected tissues. Vasodilation is induced by inflammatory mediators, such as histamine and prostaglandins, increasing blood flow to the injured site. Simultaneously, there is migration of leukocytes from the general circulation to the damaged tissue, a process mediated by chemokines. These activated leukocytes in turn produce cytokines, chemokines, and other inflammatory mediators, which further amplify the immune response. Consequently, inflammation induces an *in situ* increase in protein levels [20].

The protocol developed in this study represents a significant advancement over the methods described by Bliss *et al*. and Parkinson *et al*. through three strategic innovations: (i) an optimized lysis system using 2% SDS in 50 mM TEAB buffer, which better preserves dermal matrix protein solubility compared to urea/thiourea buffers [14] or 0.1% SDS [13]; (ii) SP3-based purification that effectively removes residual SDS, salts, and lipids, outperforming C18 cartridges [14] and protein precipitation methods; and (iii) implementation of FAIMS-DIA technology, which enhances low-abundance protein identification by overcoming limitations inherent to isobaric tagging strategies (iTRAQ) [13] while enabling comprehensive profiling of the human skin proteome.

While this study establishes a robust proteomic workflow, we acknowledge two key limitations. First, although validated using both healthy controls and sporotrichosis patients, direct application to other dermatoses with distinct histopathological features (e.g. fibrotic disorders such as scleroderma or bullous diseases like pemphigus) may require protocol optimization to address the tissue heterogeneity caused by these conditions.

Second, although our protocol achieved satisfactory proteomic coverage, analysis of whole biopsies does not discriminate the specific contribution of each skin compartment (epidermis, dermis, dermoepidermal junction region). This 'global' approach may mask critical proteomic differences between cell layers. Future developments could integrate laser microdissection techniques or prior mechanical fractionation to generate stratified proteomic maps, essential for studying localized processes or even single-cell-type protein profiling, particularly for spatially restricted processes like keratinocyte differentiation.

## Conclusion

The protocol developed for the extraction of proteins from human skin resulted in the identification of over six thousand proteins, achieving a yield three times higher compared to the protocol by Bliss *et al*., which identified approximately two thousand proteins, and ten times higher than the protocol by Parkinson *et al*., which identified six hundred proteins. These results demonstrate that our protocol, successfully optimized and adapted the process protein extraction from human skin, achieving a high yield (13, 14).

The key to achieving this outcome was optimizing protein extraction by enhancing the lysis processes and integrating chemical and mechanical techniques. As research in this area progresses, our protocol may serve as a valuable tool for future investigations into the human skin proteome.

## Supplementary Material

bpaf035_Supplementary_Data
