# Supplementary material for: Optimized protein extraction protocol from human skin samples
Source: Biol Methods Protoc. 2025 May 10;10(1):bpaf035. doi: 10.1093/biomethods/bpaf035 (PMC12202028; doi:10.1093/biomethods/bpaf035)
Supplement: bpaf035_Supplementary_Data [file bpaf035_supplementary_data.zip › Supplementary Table S1.docx]

**Table S1.** Skin protein extraction yield

| **Sample Identification** | **Total Proteins extracted (mg)/ 50 mg skin sample** | | **Protein/ Skin sample (mg)** |
| --- | --- | --- | --- |
| Control 1 | 0,73 | 0,015 | |
| Control 2 | 0,86 | 0,017 | |
| Control 3 | 1,08 | 0,021 | |
| Control 4 | 1,15 | 0,023 | |
| Control 5 | 0,83 | 0,016 | |
| Control 6 | 1,35 | 0,027 | |
| Control 7 | 1,29 | 0,026 | |
| Control 8 | 0,85 | 0,017 | |
| Control 9 | 0,79 | 0,016 | |
| Control 10 | 1,09 | 0,022 | |
| Control 11 | 1,32 | 0,026 | |
| Control 12 | 0,93 | 0,019 | |
| Patients CF 1 | 3,04 | 0,061 | |
| Patients CF 2 | 1,9 | 0,038 | |
| Patients CF 3 | 4,19 | 0,084 | |
| Patients CF 4 | 3,69 | 0,074 | |
| Patients CF 5 | 2,83 | 0,057 | |
| Patients CF 6 | 3,01 | 0,060 | |
| Patients CF 7 | 3,16 | 0,063 | |
| Patients LC 1 | 3,33 | 0,066 | |
| Patients LC 2 | 3,42 | 0,068 | |
| Patients LC 3 | 5,16 | 0,103 | |
| Patients LC 4 | 3,66 | 0,073 | |
| Patients LC 5 | 2,24 | 0,045 | |
| Patients LC 6 | 3,60 | 0,072 | |
| Patients LC 7 | 2,61 | 0,052 | |
| Patients LC 8 | 4,67 | 0,093 | |
| Patients LC 9 | 3,65 | 0,073 | |
| Patients LC 10 | 3,41 | 0,068 | |
| Patients LC 11 | 4,70 | 0,094 | |
| Patients LC 12 | 3,35 | 0,067 | |

* Legend: LC = Lymphocutaneous Sporotrichosis; CF = Fixed Cutaneous Sporotrichosis. The protein concentrations of the samples were determined using the bicinchoninic acid (BCA) method
