# Supplementary figures and images for: Optimized protein extraction protocol from human skin samples
Source: Biol Methods Protoc. 2025 May 10;10(1):bpaf035. doi: 10.1093/biomethods/bpaf035 (PMC12202028; doi:10.1093/biomethods/bpaf035)

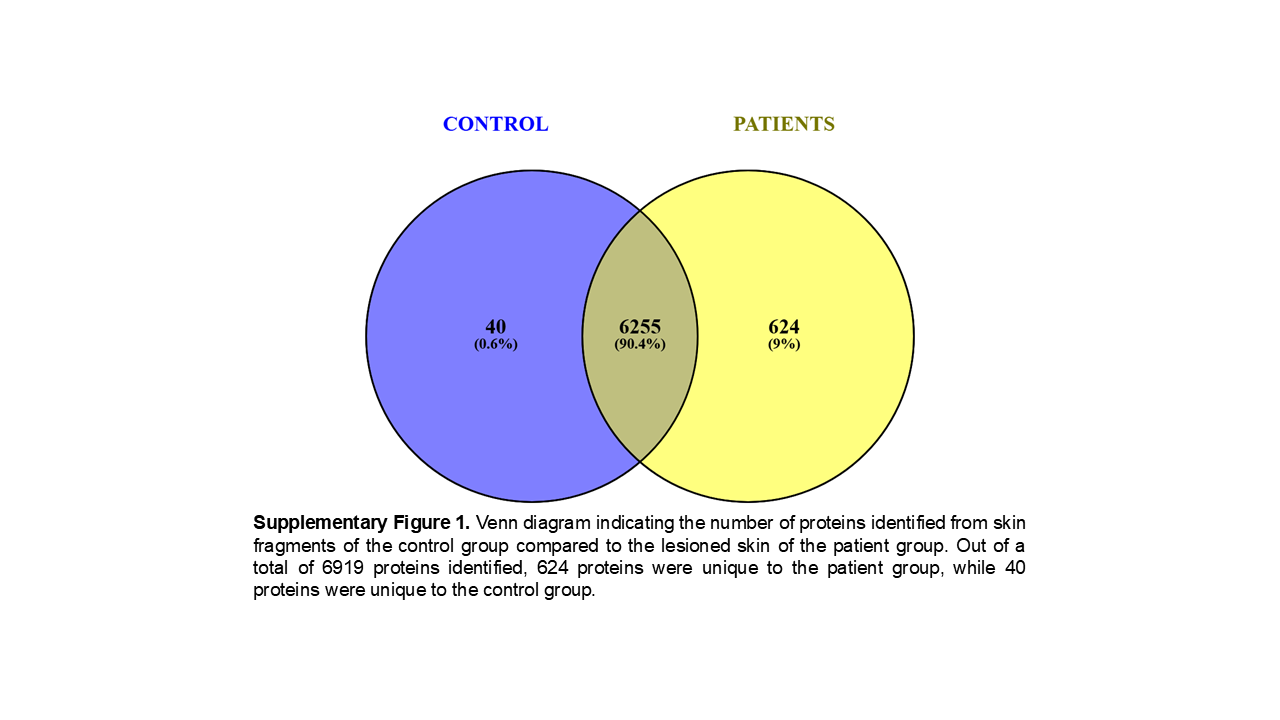

Supplement: bpaf035_Supplementary_Data [file bpaf035_supplementary_data.zip › Supplementary Figure S1.tif]
